# Enhanced Recovery of Microbial Genes and Genomes From a Marine Water Column Using Long-Read Metagenomics

**DOI:** 10.3389/fmicb.2021.708782

**Published:** 2021-08-27

**Authors:** Jose M. Haro-Moreno, Mario López-Pérez, Francisco Rodriguez-Valera

**Affiliations:** ^1^Evolutionary Genomics Group, División de Microbiología, Universidad Miguel Hernández, Alicante, Spain; ^2^Research Center for Molecular Mechanisms of Aging and Age-Related Diseases, Moscow Institute of Physics and Technology, Dolgoprudny, Russia

**Keywords:** metagenome, metagenome-assembled genomes (MAGs), long-read sequencing, PacBio CCS long-reads, polyketide synthase (PKS), CRISPR

## Abstract

Third-generation sequencing has penetrated little in metagenomics due to the high error rate and dependence for assembly on short-read designed bioinformatics. However, second-generation sequencing metagenomics (mostly Illumina) suffers from limitations, particularly in the assembly of microbes with high microdiversity and retrieval of the flexible (adaptive) fraction of prokaryotic genomes. Here, we have used a third-generation technique to study the metagenome of a well-known marine sample from the mixed epipelagic water column of the winter Mediterranean. We have compared PacBio Sequel II with the classical approach using Illumina Nextseq short reads followed by assembly to study the metagenome. Long reads allow for efficient direct retrieval of complete genes avoiding the bias of the assembly step. Besides, the application of long reads on metagenomic assembly allows for the reconstruction of much more complete metagenome-assembled genomes (MAGs), particularly from microbes with high microdiversity such as Pelagibacterales. The flexible genome of reconstructed MAGs was much more complete containing many adaptive genes (some with biotechnological potential). PacBio Sequel II CCS appears particularly suitable for cellular metagenomics due to its low error rate. For most applications of metagenomics, from community structure analysis to ecosystem functioning, long reads should be applied whenever possible. Specifically, for *in silico* screening of biotechnologically useful genes, or population genomics, long-read metagenomics appears presently as a very fruitful approach and can be analyzed from raw reads before a computationally demanding (and potentially artifactual) assembly step.

## Introduction

Metagenomics is among the most powerful tools of exploratory microbiology. Its application to several environments has allowed enlarging enormously what we know about the real (and largely unexpected) diversity of prokaryotic cells (Parks et al., 2017; Castelle and Banfield, 2018). In actuality, these advances were largely possible by the advent of high-throughput low-error short-read (SR) sequencing (such as Illumina) that has allowed the generation of enormous datasets that can be used for the assembly of composite genomes called metagenome-assembled genomes (MAGs) (Sharon and Banfield, 2013; Hugerth et al., 2015), complemented by typically incomplete and expensive to generate, but largely reliable, single-cell amplified genomes (SAGs) (Rinke et al., 2013; Pachiadaki et al., 2019). MAGs have allowed rewriting much of what we knew about microbes during the last 10 years (Chen et al., 2020). However, assembly driven metagenomics has weaknesses: (i) low recovery of high microdiversity microbes (Haro-Moreno et al., 2020); (ii) low recovery of the flexible genome (Rodriguez-Valera et al., 2009); and (iii) uncertainty due to potential chimera generation (Bowers et al., 2017).

By covering large genomic tracks, including the short- to medium-size repeats that confuse short-read assembly algorithms (Schadt et al., 2010; Goodwin et al., 2016; Pollard et al., 2018), long-read (LR) sequencing technologies (i.e., Oxford Nanopore Technologies—Nanopore, and Pacific Biosciences—PacBio) (Clarke et al., 2009; Eid et al., 2009) solve major problems for genome assembly. Thus, they allow an extremely efficient and accurate closing of viral, prokaryotic, or even eukaryotic genomes (Loman et al., 2015; Jain et al., 2018; Wenger et al., 2019; Beaulaurier et al., 2020; Bickhart et al., 2021). However, these techniques are, in general, much more prone to error than Illumina, which complicates their application for metagenomics. High coverage is a must to get a reasonably reliable sequence (Rhoads and Au, 2015). However, the recent development of PacBio Sequel II chemistry allows to significantly decrease the error rate (PacBio, 2019). Individual DNA fragments, also called subreads, are sequenced many times by the same DNA polymerase, thus allowing their overlapping into “Highly Accurate Single-Molecule Consensus Reads” (CCS reads) that share low error rates comparable to sanger and Illumina sequencing (Frank et al., 2016; Wenger et al., 2019). LR sequencing has the potential of fixing the problems of SR assembly, and it also offers a good complementarity to SAGs since it is not biased by an amplification step and is simpler and cheaper. Besides, low error-rate LR metagenomes might be annotated directly from the sequence output avoiding erroneous protein translation and call. This would allow a good metabolic reconstruction of the environment with a high-accuracy prediction of biochemical activities. The core genome, the part best reconstructed in MAGs, is often the least interesting for ecological/biotechnological applications but could be reconstructed and exploited using LR. Besides, highly reliable taxonomic affiliation by consensus similarity of multiple genes to a reference genome allows for better inference of the origin of gene clusters. Taxonomy markers such as ribosomal RNA operons can be retrieved completely allowing reliable community structure determination (Singer et al., 2016).

To assess the resolving power of PacBio Sequel II and compare it with Illumina NextSeq, we have selected an environmental metagenome rather than constructing a synthetic community (Nicholls et al., 2019; Hu et al., 2020; Moss et al., 2020) or analyzing low diversity environments (Somerville et al., 2019; Xie et al., 2020). We still do not know the real extent of the diversity of a natural complex community to be able to mimic it with mixtures of known genomes. Besides, this kind of test has already been done and provided satisfactory results (Nicholls et al., 2019; Hu et al., 2020; Moss et al., 2020). A similar analysis for a complex metagenome from terrestrial sediments has been done comparing Illumina short reads and Illumina TruSeq synthetic long reads; however, the recovery of MAGs from synthetic LR was hampered due to the relatively low throughput of the technology and the high complexity of the sample (Sharon et al., 2015). The open ocean is one of the oldest and most important communities for the global ecology of the planet and has been extensively studied by several methods, including metagenomics, for decades (Venter et al., 2004; Delong et al., 2006; Thorpe et al., 2007; Sunagawa et al., 2015). Therefore, we already have a vast amount of information to interpret the results. We took a sample from offshore Mediterranean waters in winter, when the water column is mixed, and it is likely that any depth sampled would provide a richer representation of the whole epipelagic microbiome (Haro-Moreno et al., 2018). From the same specific sampling site and season, we have abundant information from previous metagenomic analysis (Ghai et al., 2010; López-Pérez et al., 2017; Haro-Moreno et al., 2018, 2019). We applied PacBio Sequel II and analyzed the results pre- and post-assembly. We propose a specific pipeline based on CCS processing of the raw PacBio reads to retrieve more useful information directly from the individual LRs, and their assembly to provide better MAGs than SRs allow.

## Materials and Methods

### Sampling, Processing, and Sequencing

Samples from two different depths (20 and 40 m) were collected on February 15, 2019 from the epipelagic Mediterranean Sea at 20 nautical miles off the coast of Alicante (Spain) (37.35361°N, 0.286194°W) during winter where the water column is mixed. This location has been studied previously by metagenomic approaches (Ghai et al., 2010; Mizuno et al., 2013; Haro-Moreno et al., 2017, 2018, 2019; López-Pérez et al., 2017). For each depth, 200 L were collected and filtered on board as described in the study of Haro-Moreno et al. (2018). Briefly, seawater samples were sequentially filtered through 20-, 5-, and 0.22-μm pore filter polycarbonate filters (Millipore). Water was directly pumped onto the series of filters to minimize the bottle effect. Filters were immediately frozen on dry ice and stored at −80°C until processing.

DNA extraction was performed from the 0.22-μm filter (free-living bacteria) following the phenol/chloroform extraction. Given the large amount of DNA needed for sequencing, DNA from the two samples (20 and 40 m) was pooled together. Metagenomes were sequenced using Illumina Nextseq (100 bp, paired-end reads) (Macrogen, South Korea) and using PacBio Sequel II (one 8M SMRT Cell Run, 30-h movie) (Genomics Resource Center, University of Maryland, United States).

### Raw Read Filtering and Assembly of Metagenomic Samples

The quality of Illumina raw reads was examined with fastqc v0.11.9^1^. PacBio Sequel II lacked a phred score. The GC content in each sample was calculated using the gecee program from the EMBOSS v6.5.7 package (Rice et al., 2000). Illumina raw reads were trimmed with Trimmomatic v0.39 (Bolger et al., 2014) and assembled using IDBA-UD v1.1 with minimum and maximum k-mer sizes of 50 and 100, respectively, in incremental steps of 10 and the –pre_correction option activated (Peng et al., 2012), and with SPAdes (Bankevich et al., 2012) with the metagenome option and with minimum and maximum k-mer sizes of 49 and 99, respectively, in incremental steps of 10. To improve the quality of the PacBio reads, we generated Highly Accurate Single-Molecule Consensus Reads (CCS reads) using the CCS v4.2 program of the SMRT-link package. The minimum number of full-length subreads required to generate a CCS read was set to 5, 10, and 15 (99, 99.9, and 99.95 base call accuracy, respectively). PacBio (raw and CCS reads) were assembled using the following assemblers: SPAdes 3.14 (Bankevich et al., 2012) with the metagenome option and performing a hybrid assembly with the Illumina trimmed reads with minimum and maximum k-mer sizes of 49 and 99, respectively, in incremental steps of 10. CCS reads were provided as single reads (-s), whereas raw reads were provided using the –pacbio option; Flye v2.7 (Kolmogorov et al., 2020) with the metagenome option, raw reads and CCS reads were assembled individually with the –pacbio-raw and –pacbio-hifi options, respectively; and HiCanu v2.0 (Nurk et al., 2020) with default parameters and providing raw reads and CCS reads with the –pacbio and –pacbio-hifi options, respectively. MetaFlye is a *de novo* assembler that follows the classical de Bruijn graphs (DBG), although it allows for approximate sequence matches. Canu, on the other hand, applies an overlapping (OLC) strategy for *de novo* assembly. There are differences on how these two approaches (DBG and OLC) work, which have been extensively studied (Rizzi et al., 2019). OLC tends to be computationally demanding due to the fact that it performs an all-vs-all alignment of the reads to find overlapping regions and call a consensus, while DBG has a more relaxed computer requirement and therefore it has been widely used for SR assembly. Lastly, SPAdes needs both short and long reads to perform a hybrid assembly. However, in the latter case, LRs are only used for gap closure and repeat resolution. Given that the error rate in PacBio reads can be significantly improved, we used the resulting CCS15 reads as single reads in the hybrid assembly with SPAdes, and in that case, CCS reads can be used together with the Illumina reads for graph construction, gap closure, and repeat resolution.

### Taxonomic and Functional Annotation of PacBio Reads and Assemblies

Prodigal v2.6.3 (Hyatt et al., 2010) was used to predict genes from the assembled contigs retrieved from the individual assemblies of Illumina and PacBio reads, as well as from the PacBio CCS reads. tRNA and rRNA genes were predicted using tRNAscan-SE v2.0.5 (Lowe and Eddy, 1996) and barrnap v0.9^2^, respectively. Predicted protein-encoded genes were taxonomically and functionally annotated against the NCBI NR database using DIAMOND 0.9.15 (Buchfink et al., 2015) and against COG (Tatusov et al., 2001) and TIGRFAM (Haft et al., 2001) using HMMscan v3.3 (Eddy, 2011).

### Taxonomic Classification of Metagenomic Reads

16S rRNA gene sequences were retrieved from Illumina and PacBio reads. Candidate Illumina sequences in a subset of 20 million reads were extracted using USEARCH v6.1 (Edgar, 2010) after an alignment against a nonredundant version of the SILVA database v138 (Quast et al., 2013). Sequences that matched to this database with an E-value <10^–5^ were considered potential 16S rRNA gene fragments. Then, ssu-align 0.1.1 was used to identify true sequences aligning these candidate sequences against archaeal and bacterial 16S rRNA hidden Markov models (HMMs). For the long-read sequences, candidate 16S rRNA sequences were extracted using barrnap from total PacBio CCS15 reads. The resulting 16S rRNA sequences (derived from short and long reads) were classified using the sina algorithm (Pruesse et al., 2012) according to the SILVA taxonomy database. Illumina sequences were only classified if the sequence identity was ≥80% and the alignment length ≥90 bp. Sequences failing these thresholds were discarded.

Besides, a total of 170 CCS15 contigs containing 16S and 23S rRNA genes of the phylum Cyanobacteria were selected to perform an internal transcribed spacer (ITS) phylogenetic tree, using the maximum-likelihood approach in iqtree v1.6.12 (Nguyen et al., 2015), with 1,000 bootstraps and the Jukes–Cantor model of substitution. Reference cyanobacterial ITS sequences were downloaded from the NCBI database (Supplementary Table 1).

### Genome Reconstruction

Assembled contigs longer than or equal to 5 kb were assigned to a phyla classification if at least 50% of the genes shared the same best-hit taxonomy. Contigs failing this threshold were grouped as unclassified. To bin the contigs into MAGs, their taxonomic affiliation (including the unclassified) was used together with the principal component analysis of tetranucleotide frequencies, GC content, and coverage values within this sample and several metagenomic samples described in previous studies from the Mediterranean Sea (Haro-Moreno et al., 2017, 2018, 2019; López-Pérez et al., 2017). Tetranucleotide frequencies were computed using the wordfreq program in the EMBOSS package, and the principal component analysis was performed using the FactoMineR v1.42 package (Lê et al., 2008). Coverage values were calculated by the alignment of metagenomic reads (in subsets of 20 million reads) against contigs using BLASTN v2.9.0 (Altschul et al., 1997) (99% identity, >50 bp alignment). Reads were normalized by the size of the contig in kb and by the size of the metagenome in Gb (RPKGs). The degree of completeness and contamination of the resulting MAGs were estimated using CheckM v1.1.2 (Parks et al., 2015). The average nucleotide identity (ANI) between MAGs and the reference genome was calculated using the JSpecies v1.2.1 software with default parameters (Richter and Rossello-Mora, 2009).

### Retrieval of Relevant Genes From the Assemblies and the PacBio CCS Reads

Predicted protein sequences of contigs and PacBio CCS15 reads longer than or equal to 5 kb were compared against several downloaded and custom datasets. Two custom datasets of curated type-1 and type-3 rhodopsins, containing sequences from metagenomic surveys and public databases (MicRhoDE, NCBI, and UniProt) (Boeuf et al., 2015; UniProt Consortium, 2018), were constructed by aligning amino acid sequences with muscle (Edgar, 2004) with default parameters following with the construction of two HMM profiles (type-1 and type-3) using hmmbuild (Eddy, 2011). Most of the sequences used and the approach followed here have been described previously (Haro-Moreno et al., 2018; Kovalev et al., 2020; López-Pérez et al., 2020). Searches were performed using hmmscan, and only hits with an E-value <10^–20^ were considered. To remove redundant proteins, sequences were hierarchically clustered from 100% to 30% identity with decremental steps of 10% identity using cd-hit (Huang et al., 2010). Glycosyltransferases (GTs) were retrieved using dbCAN v2 (Yin et al., 2012) against the Carbohydrate-Active enZYmes (CAZy) database V8 (Lombard et al., 2014). To consider the GTs involved in the flexible genome, only genomic fragments with ≥ 5 GTs and E-values <10^–40^ were analyzed.

Lastly, the bacterial version of the secondary metabolite biosynthesis database (antiSMASH v5.1) was used to identify and classify (Blin et al., 2019) polyketide synthases (PKS) gene clusters from contigs and PacBio CCS15 reads longer than or equal to 5 kb, and their taxonomic affiliation was based on consensus, that is, >50% of proteins encoded in a contig should share the same taxonomy (see above).

### Recovery and Annotation of Novel CRISPR-cas Systems

Sequences ≥ 5 kb long were screened using CRISPR-detect v2.4 (Biswas et al., 2016) and CRISPR-cas finder v4.2.2 (Couvin et al., 2018) tools. Only sequences matching in both methods and with an evidence value ≥ 3 were kept. The taxonomical affiliation of CCS reads and assembled contigs was based on the annotation of coded proteins (>70% must share the same taxon). To find the putative target, CRISPR spacers were aligned using the blastn-short algorithm against nearly 200,000 phages collected and classified in the study of Coutinho et al. (2019). Only matches with >97% identity and 100% alignment were considered. We also expanded the search including numerous metagenomic and viromic assemblies recovered from the Mediterranean Sea (Haro-Moreno et al., 2017, 2018, 2019; López-Pérez et al., 2017) and other marine samples (Sunagawa et al., 2015; Biller et al., 2018).

### Data Availability

Metagenomic datasets have been submitted to NCBI SRA and are available under BioProject accession number PRJNA674982 (Illumina reads: MedWinter-FEB2019-I; PacBio CCS reads: MedWinter-FEB2019-PBCCS15; and PacBio Raw reads: MedWinter-FEB2019-PB). MAGs have been deposited under BioProject accession number PRJNA674982.

## Results and Discussion

### LR Platform Output

A comparison of the metagenomic datasets generated by the two sequencing platforms (Illumina, SR and PacBio, LR) is shown in Table 1. It is apparent that with equivalent costs, one PacBio run produced 18 times more raw data (Gb) than Illumina sequencing. Besides, PacBio resulted in the largest sequenced read of ca. 448.5 kb and an average read size of 5.4 kb long. PacBio Sequel II does not provide the phred quality score (base read accuracy) (Ewing and Green, 1998) of the dataset (Fukasawa et al., 2020). However, to guarantee a low error rate, we applied the software “Highly Accurate Single-Molecule Consensus Reads” (CCS reads) (Wenger et al., 2019). The algorithm selects DNA tracts that are resequenced up to a number (5, 10, or 15 times). These numbers theoretically achieve 99%, 99.9%, and 99.95% base call accuracy, respectively. Thus, for example, the total PacBio sequence generated decreased from 439.63 Gb (raw) to 7.63 Gb (CCS15) (Table 1). To assess the read accuracy, we assumed that erroneous nucleotides would lead to an increase in stop codons in the predicted proteins and could be measured by the decrease in their average protein size. As seen in Table 1, PacBio raw reads had detected error rates with an average protein size of 90.4 amino acids, while for CCS15, the average protein size was 248.4 amino acids, much closer to the expected values of the two of the dominant microbes in these waters (e.g., *Ca*. Pelagibacter HTCC7211, 302.5 or *Prochlorococcus marinus* MED4, 255). Thus, we have concluded that the quality of CCS15 reads is enough to get a reliable picture of the genes present in the sample.

**TABLE 1:** Summary statistics of the short-read and long-read sequencing technologies and protein-encoded genes retrieved from reads.

Sequencing technology	Illumina (Nextseq 2 × 100 bp)	PacBio Sequel II (8M SMRT Cell Run)			
		
Read type (processing)	Trimmed reads	Raw reads	CCS5	CCS10	CCS15
**Sequencing statistics:**					
#Sequences (millions)	234.5	81.4	2.8	1.9	1.5
#Nucleotides sequenced (Gb)	23.4	439.6	15.4	10.1	7.6
Largest read size (bp)	100	448,515	36,542	24,735	17,976
Average length read size (bp)	99.6	5,401.6	5,422.4	5,190.7	4,968.8
N50 (bp)	–	5,950	5,913	5,622	5,354
L50	–	23,013,249	960,676	665,494	534,372
**Predicted proteins (Reads >1 kb):**					
#Proteins (millions)	–	368.1	21.6	12.2	9.0
**Average protein size (aa)**	**–**	**90.4**	**195.1**	**241.5**	**248.4**
Proteins/Mb sequenced	–	837.3	1,407.1	1,212.8	1,184.4

### Taxonomic Profiling of Samples by Metagenomic rRNA Operons

**FIGURE 1:**
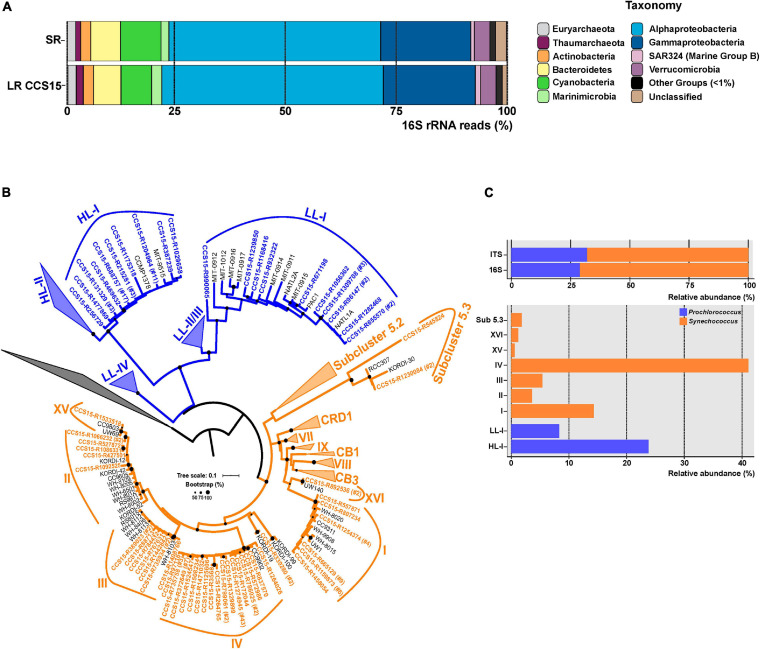
**(A)** Phylum-level composition based on 16S rRNA gene fragments of Illumina (SR) and PacBio CCS15 metagenomic reads. The phylum Proteobacteria was divided into its class-level classification. Only those groups with abundance values larger than 1% are shown. **(B)** Maximum-likelihood phylogenetic tree of the 16S–23S rRNA genes ITS extracted from CCS15 reads classified by 16S rRNA as Cyanobacteria. Sequences are colored according to their affiliation to *Prochlorococcus* (blue) or *Synechococcus* (orange). In order to simplify the tree, nucleotide sequences were dereplicated at 99% identity. Numbers between brackets indicate the number of sequences that clustered at this level to a given CCS15 read (in bold). ITS from reference isolates are also shown (black). **(C)** Upper panel, relative abundance of *Synechococcus* and *Prochlorococcus* identified from ITS or 16S rRNA sequences in the CCS15 reads. Bottom panel, ITS classified into subclades and ecotypes.

The community structure of a metagenomic sample is one of the most basic pieces of information about a microbial assemblage and can be assessed by multiple approaches (Wood and Salzberg, 2014; Huson et al., 2016). One of the most common is the retrieval of SRs that have hits to 16S rRNA genes and use their large databases to affiliate the sequences (and the microbes). This can be done with the individual SRs or with SR assembled rRNA genes (Yuan et al., 2015), although assembly of these highly conserved sequences is not very reliable. In the case of LR sequencing, complete (or nearly so) rRNA genes and even operons can be retrieved within a single read making the assembly superfluous (Benítez-Páez et al., 2016; Singer et al., 2016). We have extracted and compared 16S rRNA gene fragments to check whether LRs can improve the taxonomic affiliation. We were able to extract 9,763 16S rRNA sequences from LR CCS15 from our sample (average length: 1,207 bp; 0.34% of total LRs) and 20,564 SRs (average length: 95 bp; 0.22% of total SRs). These sequences were classified against the SILVA database (Supplementary Table 2). The community structure derived from LRs was nearly identical to the one obtained from SRs down the level of families (Figure 1A and Supplementary Table 2), with only a significant exception in Cyanobacteria, that were overrepresented in the SR dataset, 9.1% compared to 6.9% in the LRs. However, the availability of longer gene fragments with LRs improves the 16S rRNA classification, decreasing the number of reads that were not classified to any specific phylum (0.4% LR versus 1.3% SR) or could not be ascribed to lower-level taxa (for example, 4.3% only reached the class level Alphaproteobacteria with SRs versus 1.3% LRs) (Figure 1A and Supplementary Table 2). These results indicate a better resolution of LRs. More importantly, LRs have the potential to uncover complete 16S rRNA sequences from “dark matter” (Castelle and Banfield, 2018) microbes with a higher level of classification resolution and reliability by avoiding potential assembly artifacts.

Furthermore, other useful identifiers within the ribosomal operon, including hypervariable regions such as the internal transcribed spacers (ITSs), could be retrieved within a single read (Martijn et al., 2019; Okazaki et al., 2020). These allow a precise community structure determination that includes ecotypes or even strains. As an example, the ITS tree for the picocyanobacterial reads retrieved in our sample is shown in Figure 1B. We considered only complete ITS sequences (both 16S and 23S genes had to be present in the same read). A total of 170 ITSs could be extracted, of which 68% were classified as *Synechococcus*. Within this genus, clades IV (69 ITS) and I (24 ITS) were the most dominant in the sample (Figure 1C). These clades have been detected before in cold coastal waters (Zwirglmaier et al., 2007, 2008), so their presence in our mixed winter sample was expected. Along similar lines, two *Prochlorococcus* ecotypes dominated the sample. A total of 40 out of 54 ITSs were assigned to the High-Light I (HL-I) ecotype, while only 14 sequences were grouped within the Low-Light I (LL-I) ecotype (Figure 1C). These results fit well with genome recruitment data (using pure cultures or MAGs of the different ecotypes as reference) carried out on this and similar samples collected from different years, seasons, and depths (Ghai et al., 2010; Haro-Moreno et al., 2018), supporting the reliability of the LR ITS data.

### Metagenomic Assembly With LR

Still, the possibility to retrieve complete (or nearly so) genomes from metagenomes (MAGs) is highly informative for understanding uncultivated microbes. In principle, the application of LR to a complex sample could improve metagenomic assembly by simplifying the leap across repeats that hamper SR assembly. However, the choice of an assembler for metagenomic projects is not trivial. To gauge the applicability of different programs, we have to consider also the possibility of a hybrid assembly to take advantage of the high coverage and low error rate of SRs. We selected two specific assemblers for LRs (based on overlaps, Canu or de Bruijn Graphs, MetaFlye) and one that is hybrid and can combine SRs and LRs (metaSPAdes) (see the section “Materials and Methods”). Only assembled contigs larger than 5 kb have been further considered.

In a first approach, we tried to assemble the full Sequel II dataset, but the enormous number of sequences (439 Gb) was enough to collapse the assemblers. Therefore, five subsets of LRs larger than 7 kb (before CCS processing) were assembled (Supplementary Table 3). Note that the last subset (113.7 Gb) is close to the total Sequel II dataset (131.1 Gb, >7 kb reads). These five subsets were enough to evaluate the effect of different PacBio Sequel II datasets. Assembly results by metaFlye and Canu were positively correlated (close to linear, Supplementary Figure 1) with the sequencing effort. The largest contig size also followed this pattern, while the average contig size was not variable within the range of LR subsets considered. Besides, these assemblers resulted in a low number of proteins per Mb and a small average protein size, indicating error-prone assembly (Supplementary Table 3). Conversely, metaSPAdes (hybrid assembly LR and SR) showed that the effect of assembling larger amounts of PacBio raw reads did not have a linear trend in the resulting assembly (Supplementary Figure 1). Besides, due to the inclusion of SRs in the assembly process, at lower coverage values, metaSPAdes assembly was larger than the other two assemblers. Furthermore, given the restricted use of LRs in the hybrid assembly [mainly comes from the SRs (Antipov et al., 2016)], the high error rate of LRs before CCS did not significantly affect the quality (average size) of the resulting assembled proteins (Supplementary Table 3). Thus, metaSPAdes appears as the best option for the assembly of LR insofar as an SR dataset is also available.

**FIGURE 2:**
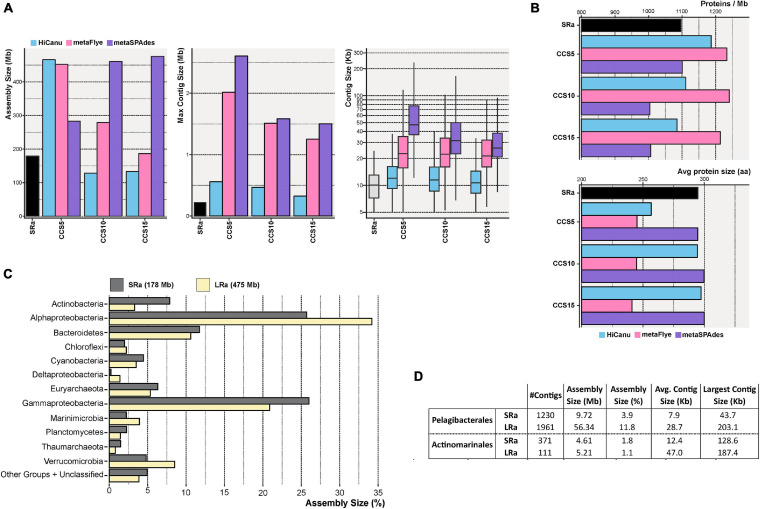
**(A)** Bar and box plots indicating the total assembly size, maximum contig length, and contig size distribution for the Illumina (SR) and PacBio CCS5, CCS10, and CCS15 assemblies. CCS reads were assembled with HiCanu, metaFlye, and metaSPAdes (blue, pink, and violet bars, respectively). **(B)** Similar to panel **(A)**, but representing the number of predicted proteins assembled per megabase (upper panel) and the average protein size (lower panel). **(C)** Taxonomic classification at the level of the phylum of the resulting Illumina (SRa) and PacBio CCS15 (LRa) assemblies. The phylum Proteobacteria was divided into its class-level classification. **(D)** Summary of the assembly statistics for contigs classified as *Ca.* Pelagibacterales and *Ca.* Actinomarinales.

We also evaluated the effect of increasing steps of CCS in the assembly by the three software packages compared to SRs IDBA assembly (SRa). Regardless of the CCS steps (5, 10, or 15), the resulting assembly outperformed IDBA SR (Figure 2A). The largest contig was achieved with metaSPAdes CCS5, 2.6 Mb long, one order of magnitude higher than the one achieved with SRa (275 kb). Besides, the average contig size was also seven times higher with metaSPAdes CCS5 (Figure 2A). Although it yielded a lower assembled output than the other two methods, the contigs had smaller average protein sizes (Figure 2B, lower panel) nullifying the longer contig advantage. Therefore, the best assembly results in terms of assembly size and, particularly, reliability were achieved using metaSPAdes with the pool of CCS15 LRs (Figures 2A,B). Unfortunately, the longest fragments obtained were shorter likely due to a decrease in the sequencing depth (Table 1). To validate the assembly of metaSPAdes CCS15 (Figure 2C), we have compared the large-scale taxonomic affiliation of the contigs with those of the SRa (Figure 2C). All phyla were recovered by both methods, and only numerical differences were found confirming that no major bias (at least not different from those that might possess SRa) was acting on the retrieval of microbial genomes by metaSPAdes CCS15 (henceforth LRa).

One major problem of the classical MAG approach is its proven low yield of some of the most prevalent members of the community. A very prominent example in the marine environment is the Pelagibacterales (Giovannoni, 2017). Despite their dominance in open epipelagic marine waters (Giovannoni, 2017), the numbers of MAGs retrieved in metagenomic studies are relatively small, with only 34 MAGs (medium quality, >50% complete, and <5% contaminated) available presently in public repositories (Haro-Moreno et al., 2020). Another example is *Ca*. Actinomarinales (Ghai et al., 2013), a cosmopolitan marine actinobacterium that accounts for up to 5% of the prokaryotic community and has only seven MAGs available (López-Pérez et al., 2020). The reasons for this anomaly are unclear, but the most likely explanation points to the high level of sequence microdiversity characteristic of these microbes. Here, the use of LR metagenomics improved considerably the assembly of contigs taxonomically affiliated to both microbes (Figure 2D). LRa achieved a better assembly size, as in Pelagibacterales, with ∼6 times more data with LRa than SRa, and longer contigs that might help the recovery of complete (or nearly so) MAGs (see below).

### Recovery of Novel Genes

One of the most useful tools of metagenomics is its application to identify new proteins that by themselves can provide important insights into the ecology of the sample and often are direct indicators of the activities of certain microbial groups. In any case, the expression in surrogate vectors allows the use of the recovered proteins for structural or biotechnological studies. LRs can span complete genes (or operons) and, therefore, avoid the SR assembly step, which is greatly biased by the choice of the assembler and gene calling tools (Hauser et al., 2016; van der Walt et al., 2017) and by the (micro)diversity and abundance of prokaryotes in the sample (Ramos-Barbero et al., 2019). A recent study (Duarte et al., 2020) evaluated how the application of high-throughput metagenomic sequencing has improved the catalog of marine microbial genes. Large metagenomic studies, such as *Tara* Oceans (Sunagawa et al., 2015), GEOTRACES (Biller et al., 2018), and Malaspina (Acinas et al., 2019), sequenced and assembled hundreds of marine SR datasets at different years, seasons, latitudes, and depths. They have retrieved ca. 50M nonredundant proteins (Duarte et al., 2020). Yet, when this number is normalized by the sequencing effort [4.8, 4.8, and 52.1 M nonredundant proteins/Tb, respectively (Duarte et al., 2020)], the numbers become smaller than those retrieved by the Global Ocean Sampling Expedition (GOS) by cloning and Sanger sequencing (Rusch et al., 2007) [624 M/Tb (Duarte et al., 2020)]. In our work, LR sequencing of just one metagenomic sample yielded 3.6M nonredundant proteins, which can be extrapolated to 473.7M/Tb, very close to the GOS numbers, but with a largely diminished cost/person-power investment and better yield of reconstructed genomes (see below) and gene clusters. To assess further the differential capability to recover novel proteins by LR metagenomics, we have selected to search in our single metagenomic sample for three common objectives of screenings for biotechnologically relevant proteins or gene clusters: rhodopsins, polyketide synthases (PKS), and CRISPR systems.

**FIGURE 3:**
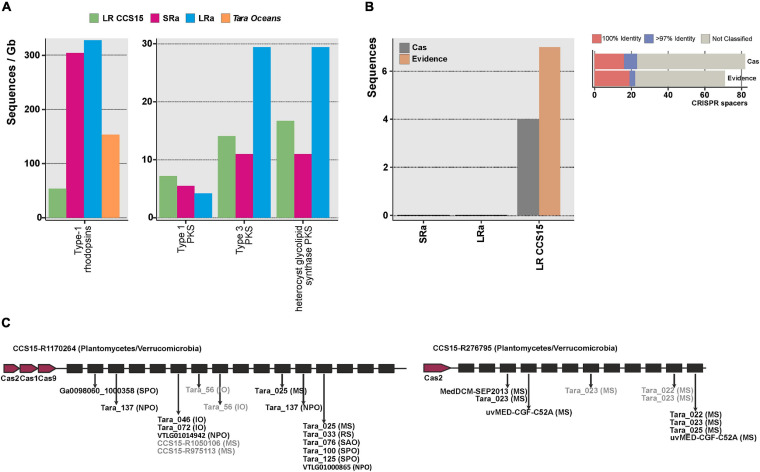
**(A)** Number of type-1 rhodopsins and polyketide synthases (PKS) (left and right panels, respectively), retrieved from the LR CCS15 reads (green bar), from SRa and LRa assemblies (red and blue bars, respectively) and from *Tara Oceans* assemblies (orange bar). Hits are normalized by the size of the database (in gigabases). **(B)** Left panel, number of sequences (LR CCS15, and SRa and LRa contigs) containing CRISPR and Cas proteins (colored in dark gray) or only a CRISPR array (Evidence) with an evidence score ≥4 (dark orange bar). Right panel, number of CRISPR spacers classified at 100 and >97% identity. Sequences failing the 97% identity threshold or not matching to the database are grouped into the “not-classified” bar. **(C)** Two examples of metagenomic reads having a CRISPR array and Cas proteins. Sequences in black and gray represent 100 and >97% identity hits. Between brackets the isolation source of the hit: Mediterranean Sea (MS); Red Sea (RS); Indian Sea (IO), North and South Atlantic Oceans (NAO, SAO); and North and South Pacific Oceans (NPO, SPO).

One of the best examples of the biotechnological harvest of metagenomics has been the retrieval of a vast diversity of retinal proteins (rhodopsins) (Béjà et al., 2000; Fuhrman et al., 2008; Pinhassi et al., 2016) critical for the development of optogenetics, a technology with remarkable potential in neurobiology and medicine (Deisseroth, 2011; Govorunova et al., 2017). The photic zone of the ocean is the quintessential habitat to screen for the diversity of rhodopsins, and already many have been retrieved by SR assembly metagenomics (Finkel et al., 2013; Bratanov et al., 2019). The largest numbers of rhodopsins (>200 amino acids, clustered at 90% amino acid identity) were found in the LRa (330 rhodopsin genes/Gb assembled) (Figure 3A). However, considering the amount of sequence assembled (31 Gb, sum of SR and LR CCS15), the relative value decreases down to 5 rhodopsins/Gb, smaller than the LR output (50 rhodopsins/Gb). This result illustrates the performance of LR to recover novel proteins avoiding the assembly step. Besides, clustering at >30% identity of all the rhodopsins retrieved with LR CCS15 and *Tara* assemblies (2,858 and 5,887 protein sequences, respectively) resulted in 25 distinct protein clusters (data not shown), 12 of them grouped sequences from both datasets. Eleven clusters had only sequences originating from *Tara* assemblies. *Tara* samples span different locations, depths, and seasons; thus, it was to be expected that its dataset contained a higher diversity of rhodopsins than our single sample. Nonetheless, we could identify three novel rhodopsin clusters not present in the *Tara* assemblies, indicating that even with a single sample, LR metagenomics could reconstruct novel rhodopsins. In fact, one of these three clusters contained sequences similar (67% amino acid identity) to RubyACRs (Govorunova et al., 2020), a recently reported anion channel rhodopsin with a promising application in optogenetics.

From a biotechnological point of view, among the most important natural products are bioactive polyketides (Miyanaga, 2017; Nivina et al., 2019). They are produced by large proteins (polyketide synthases) and often require other accompanying genes to be functional. Besides, they tend to be located at the flexible genome that, as mentioned before, assembles poorly in SR metagenomes. The total number of PKS type 1 (long and modular proteins) was similar in the three datasets (LRs, SRa, and LRa). The other two types, type 3 (smaller proteins that work only with the complement of the other members of the cluster) and heterocyst glycolipid synthase PKS (cyanobacterial PKSs), were better recovered by LRa (Figure 3A). Actually, in LR individual reads, there were type 1 complete clusters. One of them was 100% similar to the 1-heptadecene biosynthetic gene cluster from *Cyanothece* sp. PCC 7822 (Coates et al., 2014). Some type 3 PKS (mostly chalcone synthases from *Synechococcus*) were also recovered completely (data not shown). Thus, as in the case of rhodopsins, assembly can be avoided for PKSs screening from LR metagenomes.

Although CRISPR systems are very scarce in seawater (Yooseph et al., 2010), these systems are also often screened for and described from metagenomic datasets (Burstein et al., 2017). They form large clusters of Cas (CRISPR associated) proteins together with long stretches of repeats interspersed with spacers (Horvath and Barrangou, 2010). We could find four LRs containing both Cas proteins and complete CRISPR arrays (Figure 3B); this number increased up to 15 if we included sequences with no Cas proteins but with an evidence value ≥ 4 following the criterion described by CRISPRdetect on which scores above this threshold were classified as good quality based on comparison to the scores of arrays from experimentally validated species (Biswas et al., 2016). A comprehensive search of the spacers in a custom database containing metagenomes, viromes, and reference viral sequences (see the section “Materials and Methods”) showed that 28% of them were positively affiliated to viral sequences (Figure 3B). LRs CCS15-R1170264 and CCS15-R276795 represent two CRISPR arrays that are affiliated with two different and uncultured Planctomycetes/Verrucomicrobia bacteria (Figure 3C). However, their spacers indicated two different geographic distributions. Spacers in CCS15-R1170264 matched several sequences recovered from different locations, hinting at a widespread distribution of the microbe. Conversely, CCS15-R276795 indicated a possible Mediterranean endemism, since its spacers matched exclusively viral sequences recovered from metagenomes (Sunagawa et al., 2015) and fosmid libraries (Mizuno et al., 2013, 2016) from that sea.

### Recovery of Genomes

**FIGURE 4:**
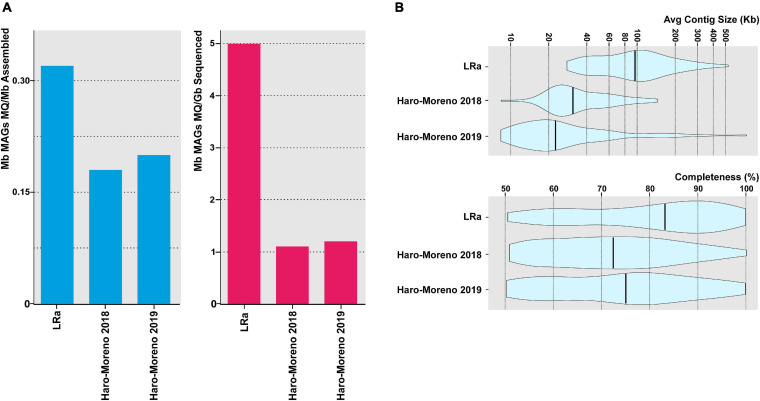
**(A)** Contribution of medium-quality MAGs (>50% complete and <5% contamination) recovered in this study (LRa) and from two previous reports from the same location in the Mediterranean Sea (Haro-Moreno et al., 2018, 2019). Values are normalized by the assembly size and sequencing effort (blue and red bars, respectively). **(B)** Violin plots showing the average contig size and the degree of completeness of MAGs described in panel **(A)**.

To assess the efficiency of MAG retrieval by LRa, we extracted 77 MAGs classified at least as of medium quality (>50% completeness, <5% contamination). This figure is rather small compared to other similar studies carried out by SRa. However, when corrected for the amount of processed sequence (CCS15 only), the ratio is higher than in similar studies carried out with similar samples by SRa, as well as the average contig size and the degree of completeness (Figure 4). To compare the MAG reconstruction carried out by both approaches, we selected 31 MAGs retrieved in the previous SRa studies carried out with similar samples (Haro-Moreno et al., 2018, 2019) and that had >99.5% average nucleotide identity (ANI) to MAGs recovered by LRa in this work (Supplementary Table 4). LRa MAGs were on average 1.5 larger than the SRa MAGs, but, even more importantly, the largest contig by LRa was 2.7 larger and the average contig size was four times larger, which switches the balance in favor of LRa for high-quality reliable MAGs.

**FIGURE 5:**
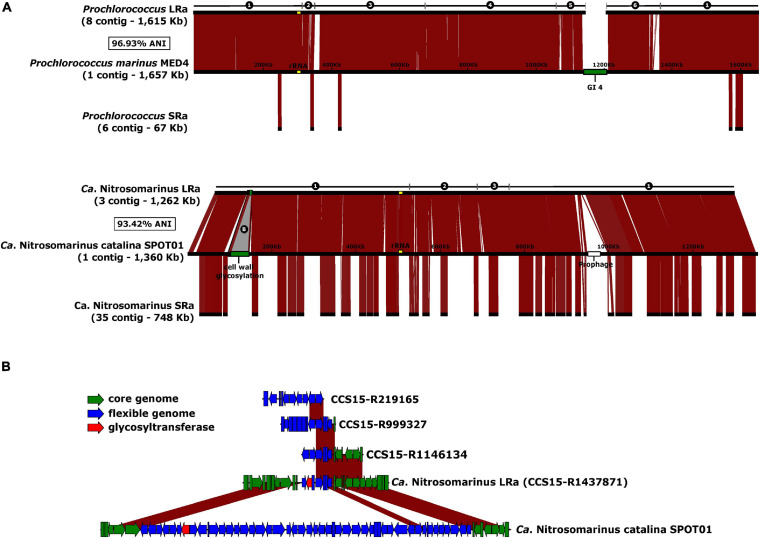
**(A)** Alignment of the reference genomes *Prochlorococcus marinus* MED4 and *Ca.* Nitrosomarinus catalina SPOT01 against the reconstructed MAGs from LRa (above) and SRa (below). Number of contigs, and ANI of the LRa MAGs and the reference genomes indicated to the left. GI4 of *P. marinus* coding for O-chain polysaccharide synthesis and a putative cell was glycosylation cluster in of *Ca.* N. catalina are in green. **(B)** Magnified B region as labeled in A indicating also some syntenic fragments found among CCS15 reads.

Visual inspection of the LRa MAGs indicated very complete and easy-to-close collections of contigs. To objectively compare the completion of MAGs generated by both approaches, we could identify two microbial genomes that are derived from pure cultures and were retrieved also in LRa and SRa, and used the culture genomes as reference (Figure 5). One of them was a genome similar (ANI 97%) to the cyanobacterium *Prochlorococcus marinus* MED4 (high-light-adapted ecotype) (Rocap et al., 2003), one of the most abundant microbes in our kind of sample (30 RPKG in the sample analyzed here). The SRa was only 2% complete (estimated by CheckM) with six small contigs among which the longest was 34 kb (Figure 5A). The LRa MAG covered nearly the complete pure culture genome with only six contigs, the longest 608 kb, and with more than 98% of the pure culture genome (Figure 5A). Gaps were found mostly at the location of the known major flexible genomic island of this microbe (Coleman et al., 2006), particularly GI4 that codes for the *O*-chain polysaccharide (Avrani et al., 2011). We also reconstructed by both assemblies a relative (93.4% ANI) of the Thaumarchaeon *Ca.* Nitrosomarinus catalina SPOT01 (Ahlgren et al., 2017). LRa produced three contigs, the longest being 1 Mb, with 99% completeness based on CheckM for archaeal conserved genes (data not shown). The comparison with the reference genome *Ca.* Nitrosomarinus catalina SPOT01 showed that only two regions were not covered (Figure 5A). One largely corresponds to a prophage that might not be present in our local relative, and the other was again a genomic island putatively involved in the synthesis of a polysaccharide that in the LRa MAG appeared much smaller.

It has been established that there are two main categories of flexible genomic islands (fGIs) in prokaryotic genomes: (i) replacement fGIs are involved in synthesizing the outer glycosidic envelope of the cells (such as the *O*-chain in Gram-negatives) (Rodriguez-Valera et al., 2016) that varies between closely related strains, and (ii) additive fGIs (such as integrons) that vary more gradually by replacement of relatively small gene cassettes that appear among other gene clusters conserved between strains. One of the problems inherent to the assembly of short reads is the failure to assemble fGIs in prokaryotic genomes. The reasons are multifactorial: (i) SRa contigs belonging to fGIs tend to bin separately due to variations in genomic parameters, (ii) they are less abundant since they are only harbored by some lineages within the population, and finally (iii) replacement fGIs tend to be surrounded by highly variable (if conserved) genes that are followed by totally divergent sequences (López-Pérez et al., 2014). All these scenarios make assembly algorithms highly inefficient in retrieving fGIs. This is a major setback for SR metagenomics since many genes of biotechnological potential are found within flexible genomic islands (Baltz, 2008; Nikolouli and Mossialos, 2012). Furthermore, many key ecological functions such as transporters, degradation of resilient compounds, virulence factors, and many others are also found in these genomic regions (López-Pérez and Rodriguez-Valera, 2016; Neuenschwander et al., 2018). That the long replacement GI4 does not assemble in the MED4 LRa MAG was to be expected given the high diversity of very polyclonal microbes such as *Prochlorococcus* (Kashtan et al., 2014) and the length of this specific fGI involved in the synthesis of the O-chain polysaccharide (Holt et al., 2020; Jayaraman et al., 2020). The presence of multiple (and long) versions of GI4 might disorient the assembler that has many possibilities to continue the contig. On the other hand, the small island present in the reconstructed *Ca*. Nitrosomarinus MAG (Figure 5B) might be short enough to appear in one single read, and it did appear in the MAG and other partial fragments recovered among the LRs but not found as SRa contigs.

To assess the improvement in the retrieval of flexible genomic islands involved in cell envelope diversity by LRa, we analyzed one type of marker that is usually found in these islands: arrays of glycosyltransferases (GTs) (Kashtan et al., 2014). Genome analyses have demonstrated that, in this GI, there is an accumulation of GTs and, therefore, these genes are a good indicator for the recovery of such gene clusters. We have considered only fragments between 5 kb encoding for at least five GTs in a window of a maximum of 20 genes as putative parts of these glycosylation islands. Indeed, LRa recovered more than 300 GT/Gb, while SRa only recovered 100. Additive flexible GIs that contain only small differential cassettes, with conserved clusters alternating with variable ones, that can be straddled by individual reads would be recovered much more efficiently, which would explain the increase in typical additive GI components such as the PKS or CRISPR (see above).

## Conclusion

This study aimed to understand whether the third-generation sequencing technology (PacBio) has addressed its characteristic high error rate and therefore was suitable for metagenomics. This study has been carried out with a single Mediterranean water column sample. The diversity of the marine pelagic prokaryotic community is very high and, although largely known at the level of phylum or even genus and species, it is still a challenge at the level of their microdiversity (Gonzaga et al., 2012; Kashtan et al., 2014) and their biotechnological exploitation. The sample selected has been studied before by multiple approaches, including rRNA cloning (Acinas et al., 1997; Zaballos et al., 2006), culture (Ivanova et al., 2015; Kimes et al., 2015), and metagenomics (Ghai et al., 2013; Martin-Cuadrado et al., 2014; Mizuno et al., 2015; López-Pérez et al., 2016; Haro-Moreno et al., 2018, 2019) except for the third generation. Furthermore, the number of genomes available as cultures or SAGs from marine water columns is also vast. Thus, we considered that it was a good choice for analyzing the performance of the new technologies in a well-known subject.

For most purposes, LR sequencing was much more rewarding both in terms of the amount and quality of the information, although SR approaches might be used to complement for recruitment of known genomes or to improve the assembly. It only requires more environmental DNA and of better quality (more care should be taken to avoid too much fragmentation of the DNA in the sample), and the cost per properly annotated gene is significantly lower. Furthermore, it allows a first glimpse at the flexible genome of many microbes in which a wealth of potentially useful biotechnology might be hidden. Previous studies using SR sequencing and assembly indicated that MAG yield does not grow linearly with sequencing effort (Rodriguez-R and Konstantinidis, 2014). We do not know whether the same trend applies for LR sequencing and assembly and this should be analyzed in future studies. Nevertheless, LRa performed better than previous Illumina assemblies, producing larger contigs that are binned more easily, and therefore, the number of MAGs recovered is higher. It might complement SAGs and MAGs to get complete and reliable genomes of the many novel groups that have been uncovered during the last decade, improve their annotation and their representation in databases, and eventually lead to a more realistic picture of the real diversity of microbes. The enhanced recovery of the flexible genome would provide a better understanding of their ecological features and their potential applications. Last but not least, the intricacy of natural populations of bacteria could be analyzed in detail providing a glimpse at microbial evolution in action.

## Data Availability Statement

The original contributions presented in the study are publicly available. This data can be found here: Metagenomic datasets have been submitted to NCBI SRA and are available under BioProject accession number PRJNA674982.

## Author Contributions

FR-V conceived the work. JH-M, ML-P, and FR-V carried out the analysis and wrote the manuscript. All authors read and approved the final version.

## Conflict of Interest

The authors declare that the research was conducted in the absence of any commercial or financial relationships that could be construed as a potential conflict of interest.

## Publisher’s Note

All claims expressed in this article are solely those of the authors and do not necessarily represent those of their affiliated organizations, or those of the publisher, the editors and the reviewers. Any product that may be evaluated in this article, or claim that may be made by its manufacturer, is not guaranteed or endorsed by the publisher.

## Footnotes

1
https://www.bioinformatics.babraham.ac.uk/projects/fastqc/

2
https://github.com/tseemann/barrnap

## Supplementary Material

Supplementary Figure 1 Comparison of the assembly size (*y*-axis) versus the assembly of five subsets of PacBio Sequel II raw reads (Supplementary Table 2). Linear trends (*y* = *mx* + *n*) are included for assemblers HiCanu and metaFlye.

Supplementary Table 1 NCBI Accession Numbers for picocyanobacterial ITS from reference genomes.

Supplementary Table 2 Relative abundance of 16S rRNA reads, based on the SILVA database.

Supplementary Table 3 Summary statistics of the assembly of PacBio raw reads using three assemblers (metaSPAdes, metaFlye, and HiCanu) and applying five read subsets.

Supplementary Table 4 Genome parameters of MAGs recovered in this study with ANI > 99.5% to MAGs retrieved from the same sampling site in the Mediterranean Sea.
